# Lead, mercury and cadmium in umbilical cord blood and its association with parental epidemiological variables and birth factors

**DOI:** 10.1186/1471-2458-13-841

**Published:** 2013-09-12

**Authors:** Esther García-Esquinas, Beatriz Pérez-Gómez, Pablo Fernández-Navarro, Mario Antonio Fernández, Concha de Paz, Ana María Pérez-Meixeira, Elisa Gil, Andrés Iriso, Juan Carlos Sanz, Jenaro Astray, Margot Cisneros, Amparo de Santos, Ángel Asensio, José Miguel García-Sagredo, José Frutos García, Jesús Vioque, Gonzalo López-Abente, Marina Pollán, María José González, Mercedes Martínez, Nuria Aragonés

**Affiliations:** 1 Environmental and Cancer Epidemiology Unit, National Centre for Epidemiology, Carlos III Institute of Health (Instituto de Salud Carlos III - ISCIII), Madrid, Spain; 2 Consortium for Biomedical Research in Epidemiology and Public Health (CIBER en Epidemiología y Salud Pública - CIBERESP), Madrid, Spain; 3 Instrumental Analysis and Environmental Chemistry Department, Organic Chemistry Institute, CSIC, Madrid, Spain; 4 Madrid Regional Health & Consumer Affairs Authority, Madrid, Spain; 5 Medical Genetics Department, Ramón y Cajal University Teaching Hospital, Madrid, Spain; 6 Public Health Department, Miguel Hernandez University, Alicante, Spain; 7 Health Prevention & Environmental Health Department, Madrid, Spain; 8 Welch Center for Prevention, Epidemiology and Clinical Research, Johns Hopkins University, Bloomberg School of Public Health, Baltimore, MD, USA

**Keywords:** Cadmium, Lead, Mercury, Biomarker, Environmental pollution, Tobacco, Pregnancy

## Abstract

**Background:**

In Spain, few studies have evaluated prenatal exposure to heavy metals. The objective of this study was to describe lead, mercury and cadmium concentrations in blood from a sample of newborn–mother-father trios, as well as to investigate the association between metals in cord blood and parental variables. We also explored the relationship between cord blood metal concentrations and child characteristics at birth.

**Methods:**

Metal correlations among family members were assessed using Spearman Rank Correlation Coefficient. Linear regression was used to explore the association between parental variables and log-transformed cord blood lead and cord blood mercury concentrations. In the case of cadmium, tobit regression was used due to the existence of samples below the detection limit. The association between cord blood metal concentrations and child characteristics at birth was evaluated using linear regression.

**Results:**

Geometric means for lead, mercury and cadmium were 14.09 μg/L, 6.72 μg/L and 0.27 μg/L in newborns; 19.80 μg/L, 3.90 μg/L and 0.53 μg/L in pregnant women; and 33.00 μg/L, 5.38 μg/L and 0.49 μg/L in men. Positive correlations were found between metal concentrations among members of the trio. Lead and cadmium concentrations were 15% and 22% higher in newborns from mothers who smoked during pregnancy, while mercury concentrations were 25% higher in newborns from mothers with greater fish intake. Cord-blood lead levels showed seasonal periodicity, with lower concentrations observed in winter. Cord blood cadmium concentrations over 0.29 μg/L were associated with lower 1-minute and 5-minute Apgar scores.

**Conclusions:**

These results reinforce the need to establish biomonitoring programs in Spain, and provide support for tobacco smoke and fish consumption as important preventable sources of heavy metal exposure in newborns. Additionally, our findings support the hypothesis that cadmium exposure might be deleterious to fetal development.

## Background

Heavy metal exposure during pregnancy is potentially harmful to the developing fetus. Lead and methylmercury easily cross the placenta and the fetal blood-brain barrier, and can irreversibly affect cognitive development [1-5]. Lead exposure can also cause spontaneous abortions [6], congenital malformations [7], reduced birth weight [8] and length [9], gestational hypertension [10] or impaired neurodevelopment [11]. Occupational exposure to mercury has been associated with pregnancy-induced hypertension [5], low birth weight [12] and birth defects [13]. Cadmium exposure is also of great concern, due to its possible effects on foetal health. Although the placenta acts as a barrier, protecting the foetus from cadmium exposure by increasing metallothionein expression [14], this metal can be found in cord blood and has been associated with decreased birth weight [15], premature delivery [16] and altered thyroid hormone status of newborns [17].

Exposure to mercury mainly occurs through ingestion of contaminated fish (methylmercury) [18] or dental amalgams (inorganic mercury) [19]. Sources of lead include leaded gasoline, lead paint hazards (including lead in paint, dust and soil), water carried out in lead pipes, industrial emissions or occupational exposures [20]. Because lead is accumulated in maternal bones and released into blood during pregnancy [21], blood lead in pregnant women can be indicative of current or past maternal lead exposure. Environmental cadmium pollution is ubiquitous owing to industrial activities, use of phosphate fertilisers, combustion of motor fuels in vehicles and particles released by tyre wear, all of which result in emissions to air, soil and water [22-24]. In non-smokers diet is the most important source of cadmium exposure. In smokers, tobacco is the most important source, since tobacco, like other plants, takes up cadmium, which is afterwards inhaled in the smoke [25].

This report is part of the BioMadrid Project, a cross-sectional biomonitoring study designed to assess environmental exposure to different pollutants in children born in the Madrid Autonomous Region, and their parents. We here present the first Spanish report on heavy metal concentrations in blood samples from newborns, pregnant women and their partners. BioMadrid allows studying the association between certain epidemiological maternal factors and cord blood metals’ concentration. We also explore the possible relationship between cord blood metals and certain birth factors (gestational age, birth weight and length, 1- and 5-minute Apgar scores).

## Methods

### Subjects and data-collection

The BioMadrid Project was a biomonitoring pilot study covering a sample of father-pregnant woman-newborn trios residing in two areas of the Madrid Autonomous Region, namely, a municipal district in the city of Madrid (urban area) and a second zone lying in the Greater Madrid Metropolitan Belt (metropolitan area). These two areas, similar in terms of population size, are representative of the two main types of urban environments in the region. The project recruited 145 triplets. Both the design and field work have been described in detail elsewhere [26]. Briefly, all pregnant women residing in the study areas and attending childbirth preparation classes in the public health care system were invited, along with their respective partners, to take part in the study, until the selected sample size was attained. Recruitment lasted from October 2003 to May 2004. No exclusions were made in terms of parents’ or newborns’ disease, ethnicity or place of origin. For logistical reasons, women were required to be aged over 15 years, to be expecting a single pregnancy, and to intend to deliver their babies at the public hospital assigned to them. Exclusions were made if they had received any blood transfusions in the previous year or if they had resided for less than one year in the study area.

Midwives scheduled a date for both parents to collect blood samples (30 ml), coinciding with the programmed control of the pregnant women and supplied the mothers a newborn sampling kit, which included pre-labeled tubes for cord-blood collection. Trained personnel interviewed both parents at their home to complete both an epidemiological questionnaire (socio-demographic factors, environmental and occupational exposures. In addition, they also filled a previously validated food-frequency questionnaire (FFQ)). The FFQ, a modified version of the Harvard questionnaire adapted for use in adult Spanish populations, was used to assess the usual dietary intake over the previous months [27]. The nine possible answers range from “never or less than once a month” to “six or more times per day”. Intake frequencies are combined with standard portion sizes and converted into average daily intake for each food group and participant. In 135 couples we had complete information, with blood samples, epidemiological and dietary questionnaire from both parents.

At delivery, mothers should give the midwives the newborn kit. If feasible, a specimen of cord-blood (12.5 ml) was drawn immediately after birth (N = 114). Logistical problems (births at non-participant hospitals due to overcrowded maternal wards in the reference centers, mothers who forgot the kit, problems to draw blood from the umbilical cord vein and samples stored incorrectly) or problems at delivery (one child was born dead and in three cases an emergency cesarean section was needed), precluded the availability of cord blood in the other trios.

During hospitalization, a protocoled clinical examination of the newborns was performed. Anthropometric data were measured once, before breastfeeding started. Infants were weighed without diapers and using an electronic digital infant scale. Length was measured in the supine position, using a stadiometer composed of a stationary head-board and a movable footboard. Knees and hips were extended using gentle force and the footboard pressed against the balls of the feet. The Apgar score was measured on a scale from 1 to 10, at 1 and 5 minutes after delivery. Infants were evaluated on a scale of 0 to 2 according to five categories (skin color, muscle tone, reflexes, respiratory effort and heart rate), and the points from each category added together to determine the total score.

In this study we first described metal levels in all BioMadrid participants with blood samples available (140 mothers, 140 fathers and 114 children). We then excluded trios with no cord-blood samples available or with problems at delivery, trios where at least one member had no metal determinations, and trios where at least one adult had not completed the epidemiological questionnaire, leading to a final sample of 112 complete trios. Finally, in the sub-sample of trios with all information available, we evaluated the possible associations between parental factors, umbilical cord blood metal levels and newborn’s characteristics at birth.

### Laboratory

Total lead and cadmium concentrations were determined using a Perkin Elmer Analyst 600 Atomic Absorption Spectrometer (Perkin Elmer Hispania, Madrid, Spain), fitted with a transversely heated graphite atomizer furnace assembly and longitudinal Zeeman-effect background correction. Mercury was measured by a Perkin Elmer FIMS-400 Atomic Absorption Spectrometer (Perkin Elmer Hispania, Madrid, Spain) using the cold vapor technique (Cold Vapor Atomic Absorption Spectrometer). We used mercury as a reliable measure of methylmercury because it has been reported that when mercury concentrations are as high as those found in our study, methylmercury accounts for up to 90% of mercury [28].

Detection limits were as follows: 1.70 μg/L for lead, 0.12 μg/L for mercury and 0.25 μg/L for cadmium. For lead and mercury all values exceeded the limit of detection. For cadmium, 47% of samples in cord blood and 15% of samples in peripheral blood had concentrations below 0.25 μg/L. Lyophilised control material SeronormTM (Trace Elements Whole Blood 2, SERO, Billingstand, Norway) was used to verify the precision and accuracy of the analytical measurements. The coefficients of variation for the three metals were less than 5%. In all cases, analyses were performed after the correct values had been confirmed by the instrumentation.

### Statistical analysis

Cadmium values below the detection limit were replaced by half the detection limit for statistical analysis [29]. Differences in the distribution of heavy metals between samples were visually examined using box-plots. Spearman test was used to assess metals’ correlations among members of the trio.

Geometric means and 95% confidence intervals, as well as 25^th^, 50^th^ and 75^th^ percentiles, were calculated for the three metals in maternal, paternal and umbilical cord blood. Also, geometric means and 95% confidence intervals were computed for cord blood concentrations by parental characteristics. For lead and mercury, the ratio of geometric means across categories of parental characteristics was estimated by fitting multivariable linear regression models, introducing the log-transformed metal concentration as the dependent variable and adjusting for potential confounders previously described in the literature: parental age (continuous), living region (metropolitan/urban) and educational level (< high school/high school/>high school). Mercury models were also adjusted for fish consumption (grams/day). In a second step, lead models were further adjusted for cord-blood sampling season according to date of birth and for maternal tobacco smoke, with similar results (data not shown). The estimated beta coefficients and standard errors of the models were exponentiated to show the results in a more easily understandable way.

Due to the number of samples with concentration of cadmium below the detection limit, Tobit regression models were fitted to explore differences in cadmium concentrations according to maternal or paternal characteristics. In this method, linear regression is applied to non-censored continuous data, conditional on an assumed influence on censored data, and although the coefficients can be interpreted in similar manner to what we have explained for lineal regression models, caution should be taken as the lineal effect is on the uncensored latent variable and not on the observed outcome [30]. Tobit models were also adjusted for potential confounders: age, living region, educational level and parental smoking habits (never-smoked/quitted before pregnancy/smoked during pregnancy).

Finally, multivariable lineal regression models were fitted to study the relationship between cord blood metal levels and newborn’s anthropometric measures and Apgar scores. For this purpose, cord blood metal concentrations were dichotomized at the median (13.8 μg/L for lead, 7.7 μg/L for mercury, and 0.29 μg/L for cadmium). These models were adjusted for maternal age, newborn’s sex and gestational age, since these factors are known to influence birth outcomes; as well as for those variables associated with metal levels in our population (maternal tobacco smoking and sampling season for lead, fish consumption for mercury and maternal tobacco smoking for cadmium).

### Ethical considerations

All participants signed an informed consent document which included information on their statutory rights to confidentiality and protection of personal data. The study protocol was formally approved by the Ethics Committee of the Carlos III Institute of Health, in accordance with the principles of the Helsinki Declaration.

## Results

Table 1 presents the main descriptive statistics for lead, mercury and cadmium in all study participants. Lead and cadmium levels were lower in umbilical cord blood than in parental blood, while mercury levels showed the highest concentrations in children. In fathers, geometric mean lead and mercury levels were higher than those of mothers.

**Table 1:** Heavy metal levels in all BioMadrid participants with blood samples and metal determinations available

**Metal**	**Specimen**	**Participant**	**N**	**Geometric mean (95% CI)**	p25**	**p50**	**p75**
**Lead (ug/L)**	**Umbilical cord blood**	Newborn	114	14.09 (12.77-15.55)	10.56	13.80	19.11
	**Peripheral blood**	Women	140	19.80 (18.16-21.62)	14.08	18.98	27.21
	**Peripheral blood**	Men	140	33.00 (30.48-35.64)	23.81	33.24	43.21
**Mercury (ug/L)**	**Umbilical cord blood**	Newborn	108*	6.72 (5.74-7.87)	4.95	7.66	11.03
	**Peripheral blood**	Women	140	3.90 (3.38-4.50)	2.39	4.61	6.83
	**Peripheral blood**	Men	140	5.38 (4.69-6.17)	3.46	5.99	9.13
**Cadmium (ug/L)**	**Umbilical cord blood**	Newborn	114	0.27 (0.23-0.31)	0.13	0.27	0.55
	**Peripheral blood**	Women	140	0.53 (0.45-0.61)	0.31	0.60	0.96
	**Peripheral blood**	Men	140	0.49 (0.42-0.58)	0.13	0.63	1.09

*p25 = percentile 25^th^; p50 = percentile 50^th^; p75 = percentile 75^th^.

**In six cases, cord blood samples were insufficient for mercury analyses.

Significant positive correlations were found between lead, mercury and cadmium concentrations in maternal and umbilical cord blood (r_Pb=_0.40, p < 0.001; r_Hg=_0.57, p < 0.001; r_Cd_ = 0.25, p < 0.001), and between lead and mercury concentrations in paternal and umbilical cord blood (r_Pb=_0.27, p < 0.001; r_Hg=_0.43, p < 0.001). Metal concentrations were also correlated among parents (r_Pb_ = 0.27, p < 0.001; r_Hg_ = 0.63, p < 0.05 and r_Cd_ = 0.37, p < 0.001). Interestingly, no correlation was seen between cadmium concentrations in paternal and umbilical blood.

Tables 2 and 3 present cord blood metal levels by parental characteristics in those children for which both parents provided epidemiological data. The mean age was 31.1 years for mothers and 32.0 for fathers. Regarding ethnicity, 76% of women and 84% of men were Caucasians. Place of residence was almost equally divided between the two study areas and participants reported living in the same home for an average of 10.2 years (95% CI: 8.79-11.62). While approximately 61% of women and 69% of men were passively exposed to tobacco smoke in the workplace or at home, 16% of women and 33% of men actively smoked during pregnancy. Among smokers, the median number of cigarettes smoked during pregnancy was 4 cig/day (SD: 2.8) for women, and 14 cig/day (SD: 8.4) for men. Pregnant women and their partners consumed a median of 76 and 79 grams of fish per day, respectively. No parents were occupationally exposed to mercury.

**Table 2:** Lead, mercury and cadmium concentrations (μg/L) in umbilical cord, overall and by maternal epidemiological characteristics

**Variable**	**Lead**						**Mercury**						**Cadmium**					
	**Descriptive analysis by maternal characteristics**			**Regression adjustment for maternal factors**			**Descriptive analysis by maternal characteristics**			**Regression adjustment for maternal factors**			**Descriptive analysis by maternal characteristics**			**Regression adjustment for maternal factors**		
	**N**	**GM**	**CI95%**	**e**^**β**^	**CI95%**	**p**	**N**	**GM**	**CI95%**	**e**^**β**^	**CI95%**	**p**	**N**	**GM**	**CI95%**	**e**^**β**^	**CI95%**	**p**
**Overall**	112	14.1	28.5-34.1				106	6.76	5.74-7.90				112	0.27	0.23-0.31			
**Socio-demographic factors**																		
Age																		
<=30	54	13.6	11.8-15.6	1.00			50	5.98	4.59-7.78	1.00			54	0.26	0.30-0.32	1.00		
>30	58	14.6	12.7-16.8	1.04	0.95-1.12	0.41	56	7.49	6.17-9.09	1.12	0.99-1.25	0.10*	58	0.28	0.23-0.34	1.02	0.90-1.16	0.72
Area (district)																		
Metropolitan	61	13.6	11.8-15.6	1.00			56	6.59	5.29-8.22	1.00			61	0.26	0.21-0.32	1.00		
Urban	51	14.8	12.7-17.1	1.04	0.95-1.12	0.43	50	6.90	5.42-8.78	1.03	0.89-1.17	0.70	51	0.29	0.23-0.35	1.15	0.99-1.33	0.08
Ethnicity																		
Caucasian	85	13.8	12.2-15.6	1.00			78	6.05	4.91-7.44	1.00			85	0.25	0.21-0.30	1.00		
Other	27	15.1	12.8-17.8	1.03	0.93-1.14	0.50	28	9.22	7.94-10.7	0.92	0.75-1.08	0.32	27	0.33	0.23-0.45	0.99	0.95-1.04	0.82
Educational level																		
< High school	32	15.3	12.9-18.1	1.00.			31	6.14	4.39-8.58	1.00			32	0.25	0.19-0.34	1.00		
High School	43	14.4	12.0-17.3	0.97	0.87-1.08		39	6.49	4.81-8.76	0.98	0.83-1.16		43	0.24	0.19-0.32	0.98	0.84-1.14	
>High school	37	12.8	10.8-15.2	0.91	0.81-1.02	0.11	36	7.59	6.13-9.41	1.09	0.91-1.30	0.80	37	0.31	0.25-0.40	1.11	0.94-1.31	0.20
**Obstetric history**																		
Previous pregnancies																		
None	49	13.4	11.6-15.4	1.00			46	6.66	5.13-8.66	1.00			49	0.27	0.21-0.34	1.00		
One	24	12.6	9.6-16.4	0.92	0.82-1.04		23	7.48	5.08-11.0	0.94	0.78-1.14		24	0.29	0.21-0.39	1.06	0.89-1.26	
More	13	14.6	10.6-20.2	0.99	0.86-1.15	0.59	13	6.43	4.03-10.3	0.89	0.70-1.12	0.29	13	0.26	0.15-0.43	0.94	0.75-1.17	0.80
Previous abortions																		
No	84	13.8	12.2-15.5	1.00			78	6.57	5.39-8.02	1.00			84	0.27	0.22-0.32	1.00		
Yes	28	15.1	12.5-18.4	1.04	0.94-1.15	0.42	28	7.21	5.54-9.38	0.98	0.84-1.15	0.83	28	0.27	0.20-0.38	1.00	0.86-1.17	0.94
Previous lactations																		
>6 months	10	11.4	8.12-15.9	1.00			10	4.80	2.10-11.0	1.00			10	0.35	0.21-0.60	1.00		
≤6 months	11	14.2	8.28-24.3	1.06	0.87-1.29	0.55	10	7.33	4.20-12.8	1.09	0.80-1.50	0.58	11	0.29	0.16-0.53	0.92	0.69-1.21	0.53
**Tobacco exposure**																		
Active smoking																		
Never	53	12.7	11.2-14.4	1.00.			51	6.57	5.11-8.44	1.00			53	0.23	0.19-0.28	1.00		
Former	41	14.7	12.2-17.9	1.06	0.96-1.17		39	6.88	5.43-8.72	1.00	0.86-1.16		41	0.28	0.22-0.37	1.09	0.95-1.25	
Current	18	17.3	13.4-22.3	1.15	1.02-1.31	0.02**	16	6.92	4.26-11.2	0.98	0.80-1.21	0.10*	18	0.36	0.34-0.56	1.22	1.02-1.46	0.03**
Passive smoking																		
No	83	12.9	11.4-14.7	1.00			58	6.40	5.10-8.02	1.00			83	0.26	0.22-0.31	1.00		
Yes	29	15.6	13.3-18.2	1.00	0.99-1.17	0.08*	48	7.16	5.68-9.03	1.02	0.87-1.17	0.79	29	0.30	0.22-0.41	1.03	0.88-1.21	0.70
**Fish intake (tertiles)**																		
1^st^: <=60 g/day							38	5.18	4.03-6.66	1.00			38	0.29	0.21-0.37	1.00		
2^nd^: 60-100 g/day							34	7.12	4.96-10.2	1.17	0.99-1.38		34	0.28	0.21-0.36	0.95	0.80-1.13	
3^rd^: >100 g/day							34	8.54	6.99-10.4	1.25	1.06-1.48	0.03**	34	0.24	0.19-0.32	0.90	0.75-1.09	0.28
**Amalgams use**																		
Yes							76	6.88	5.73-8.27	1.00								
No							6	6.35	1.27-31.9	0.89	0.58-1.21	0.50						
**Birth season**																		
Fall/Spring	29	15.9	13.4-19.0	1.00			28	5.09	3.42-7.58	1.00			29	0.24	0.28-0.33	1.00		
Winter	64	12.7	11.1-14.5	0.91	0.82-1.00	0.02**	59	7.33	5.95-9.05	1.06	0.92-1.22	0.40	64	0.27	0.22-0.33	1.00	0.88-1.15	0.94

**Descriptive analysis: N**: Sample size; **GM**: Geometric mean; **CI 95%:** 95% confidence interval for geometric mean * p-value < 0.10; **p-value <0.05.

**Regression analysis:** Lead models were adjusted for maternal age (continuous), living region (metropolitan,urban) and educational level (< High school, High School, >High school); Mercury models were adjusted for maternal age (continuous), living region (metropolitan/urban), educational level (< High school, High School, >High school) and tertiles of fish intake (grams/day). Cadmium models were adjusted for maternal age (continuous), living region (metropolitan/urban), educational level (< High school, High School, >High school) and tobacco smoke (never/former/current). **P:** p-value for linear trend using the explanatory variable as a continuous term.

**Table 3:** Lead, mercury and cadmium concentrations (μg/L) in umbilical cord, overall and by paternal epidemiological characteristics

**Variable**	**Lead**						**Mercury**						**Cadmium**					
	**Descriptive analysis by paternal characteristics**			**Regression adjustment for paternal factors**			**Descriptive analysis by paternal characteristics**						**Regression adjustment for paternal factors**					
	**N**	**GM**	**CI95%**	**e**^**β**^	**CI95%**	**p**	**N**	**GM**	**CI95%**	**e**^**β**^	**CI95%**	**p**	**N**	**GM**	**CI95%**	**e**^**β**^	**CI95%**	**p**
**Overall**	112	14.1	28.5-34.1				106	6.76	5.74-7.90				112	0.27	0.23-0.31			
**Socio-demographic factors**																		
Age																		
<=30	34	13.5	11.1-16.4	1.00			32	6.14	4.57-8.25	1.00			34	0.28	0.21-0.39	1.00		
>30	78	14.4	12.8-16.2	1.03	0.93-1.14	0.53	74	7.01	5.77-8.51	1.02	0.87-1.20	0.80	78	0.26	0.22-0.31	0.91	0.79-1.04	0.17
Area (district)																		
Metropolitan	61	13.6	11.8-15.6	1.00			56	6.59	5.28-8.21	1.00			61	0.26	0.21-0.32	1.00		
Urban	51	14.8	12.7-17.1	1.03	0.95-1.13	0.47	50	6.90	4.42-8.78	1.02	0.88-1.17	0.81	51	0.28	0.23-0.35	1.08	0.95-1.23	0.24
Ethnicity																		
Caucasian	94	14.1	12.6-15.8	1.00			88	6.43	5.32-7.77	1.00			94	0.26	0.22-0.31	1.00		
Other	15	13.4	10.8-16.5	0.98	0.86-1.12	0.78	15	8.47	7.04-10.2	1.11	0.91-1.37	0.30	15	0.30	0.18-0.51	0.99	0.81-1.20	0.90
Educational level																		
< High school	40	14.7	12.3-17.6	1.00			37	5.62	4.14-7.63	1.00			40	0.22	0.18-0.29	1.00		
High School	46	13.5	11.5-15.6	0.96	0.86-1.06		44	8.61	7.00-10.6	1.21	0.98-1.46		46	0.31	0.25-0.39	1.16	0.99-1.33	
>High school	26	14.2	11.6-17.3	0.98	0.87-1.10	0.65	25	6.82	5.21-8.91	1.01	0.96-1.13	0.42	26	0.26	0.19-0.36	1.12	0.95-1.34	0.14
**Tobacco exposure**																		
Active smoking																		
Never	55	13.4	11.7-15.3	1.00			54	6.64	5.37-8.20	1.00			55	0.25	0.20-0.30	1.00		
Former	18	14.8	10.9-19.9	1.05	0.92-1.20		16	9.12	6.44-12.9	1.15	0.93-1.41		18	0.28	0.18-0.45	1.09	0.91-1.30	
Current	36	14.8	12.2-18.0	1.05	0.94-1.17	0.35	33	5.70	4.02-8.08	0.97	0.82-1.15	0.83	36	0.28	0.21-0.36	1.11	0.96-1.29	0.15
Passive smoking																		
No	35	12.6	10.6-14.9	1.00			35	6.97	5.38-9.03	1.00			35	0.28	0.21-0.37	1.00		
Yes	77	14.8	13.1-16.8	1.07	0.97-1.15	0.27	71	6.62	5.39-8.13	0.99	0.86-1.16	0.96	77	0.26	0.22-0.31	1.01	0.88-1.17	0.81
**Fish intake (tertiles)**																		
1^st^: <=60 g/day							38	5.24	3.77-7.29	1.00			40	0.25	0.20-0.33	1.00		
2^nd^: 60-100 g/day							33	7.58	5.77-9.98	1.18	1.00-1.40		35	0.26	0.20-0.33	1.04	0.89-1.21	0.63
3^rd^: >100 g/day							34	8.00	6.47-9.91	1.21	1.03-1.43	0.02**	36	0.30	0.23-0.40	1.10	0.95-1.29	0.20
**Amalgams use**																		
Yes							73	6.99	5.41-8.93	1.00								
No							8	6.53	5.43-9.03	0.99	0.87-1.15	0.93						

**Descriptive analysis: N**: Sample size; **GM**: Geometric mean; **CI 95%:** 95% confidence interval for geometric mean; **p-value <0.05.

**Regression analysis:** Lead models were adjusted for paternal age (continuous), living region (metropolitan,urban) and educational level (< High school, High School, >High school); Mercury models were adjusted for paternal age (continuous), living region (metropolitan/urban), educational level (< High school, High School, >High school) and tertiles of fish intake (grams/day). Cadmium models were adjusted for paternal age (continuous), living region (metropolitan/urban) and tobacco smoke (never/former/current). **P:** p-value for linear trend using the explanatory variable as a continuous term.

After multivariable adjustment, blood lead and cadmium concentrations were 15% and 22% higher in cord blood from newborns whose mothers smoked during pregnancy. Children whose mothers were passively exposed to tobacco smoke also presented higher blood lead concentrations, although the difference was not statistically significant. In addition, cord-blood lead levels were lower among children born in winter (data not shown). Mercury concentrations were 25% higher in cord blood from mothers with greater fish intake (>100 g/day) (Table 2). Similarly, paternal fish consumption was associated with mercury levels in newborns; while fish intake showed a strong correlation among parents (r = 0.59, p < 0.01). No other paternal factors were associated with cord blood metals’ concentrations (Table 3).

Mean gestational age was 39.1 weeks and 5 children were born prematurely (<37 weeks). Mean weight at birth was 3282 grams. All children had Apgar scores over 5. Newborns with cadmium concentrations over 0.29 μg/L had lower 1-minute and 5-minute Apgar scores compared to those with lower concentrations (Table 4). No associations were observed between metal levels and newborn’s weight or length.

**Table 4:** Mean (SD) birth weight, birth length, 1-minute and 5-minutes Apgar scores, and their association with umbilical cord blood mercury, lead and cadmium levels

		**Weight (grams)**		**Length (centimeters)**		**1-minute Apgar score**		**5-minutes Apgar score**	
		**Mean (SD)**	**β (95% CI)**	**Mean (SD)**	**β (95% CI)**	**Mean (SD)**	**β (95% CI)**	**Mean (SD)**	**β (95% CI)**
**Lead**									
<=13.8		3,264 (462)	Ref.	49.8 (2.4)	Ref.	8.21 (1.4)	Ref.	9.20 (0.7)	Ref.
>13.9		3,298 (444)	123 (-37.9,284)	49.8 (2.3)	0.52 (-0.39,1.44)	8.70 (1.0)	0.67 (-0.19,1.16)	9.40 (0.6)	0.29 (-0.04,0.54)
**Mercury**									
<=7.7		3,297 (500)	Ref.	49.7 (2.4)	Ref.	8.64 (1.0)	Ref.	9.38 (0.7)	Ref.
>7.8		3,268 (402)	22.1 (-148,192)	49.9 (2.3)	0.57 (-0.32,1.46)	8.31 (1.3)	-0.31 (-0.81,0.20)	9.22 (0.6)	-0.11 (-0.37,0.16)
**Cadmium**									
<=0.29		3,381 (462)	Ref.	50.2 (2.3)	Ref.	8.70 (1.1)	Ref.	9.40 (0.5)	Ref.
>0.30		3,187 (422)	-152 (-311,7.42)	49.5 (3.3)	-0.50 (-0.41,0.41)	8.18 (1.2)	-0.57 (-1.06,-0.08)**	9.13 (0.6)	-0.24 (-0.60,-0.09)**

**p-value <0.05.

Blood metal concentrations were dichotomized at the median (13.8 μg/L for lead, 7.7 μg/L for mercury and 0.29 μg/L for cadmium). All models were adjusted for newborn’s sex, gestational age and maternal age. Lead models further adjusted for maternal cigarette smoking and sampling season. Mercury and cadmium models further adjusted for maternal fish consumption and cigarette smoking, respectively.

## Discussion and conclusion

In Spain, few studies have evaluated prenatal exposure to heavy metals. Our results are the first to provide information on blood metal concentrations in a sample of father-mother-newborn trios in the Madrid Autonomous Region, and can serve as a reference for monitoring metal levels in our environment.

The cord blood lead levels found in our study were similar to those observed in newborns from other European countries during the same period [31,32], with only one child showing lead concentrations above the present CDC reference level of 50 μg/L (CDC., 2012). A previous study in the Spanish city of Barcelona showed a reduction on cord blood lead concentrations from 186 μg/L in 1984 to 40.06 μg/L in 1995, parallel to the decrease in lead petrol concentrations in the ambient air [33]. Umbilical cord lead concentrations from our study are consistent with this dramatic decrease and reflect the success of banning leaded gasoline in Spain. Moreover, a recently published study analysing lead concentrations in cord samples collected between 2004 and 2008 in 4 different Spanish cities found a mean concentration of 10 μg/L, suggesting that blood lead levels are still declining in our country [34].

For mercury, more than 70% of our children exceeded the concentration of 5.8 μg/L which has been established by the Environmental Protection Agency (EPA) as associated with possible health effects [35]. Similar findings have been previously described in other Mediterranean countries including Spain [36] and are attributed to maternal consumption of fish species high in mercury. Regarding cadmium, though its levels were similar to those reported by other European countries during the same period [31,37,38], 29% of children surpassed the reference level of 0.5 μg/L established by the Human Biomonitoring Commission of the German Federal Environmental Agency for children [39].

In fathers, blood lead and cadmium levels were comparable to those found in men from the general population in Germany [40] or France [41] and can be considered relatively low. In this regard, no men showed blood cadmium levels above the limit of 5 μg/L established by the federal Occupational Safety and Health Administration (OSHA) in the United States [42], and only one presented blood lead levels above 100 μg/L. Conversely, mercury concentrations were considerably high [43,44]. When referring to pregnant women, inter-study comparisons were limited by the relative scarcity of studies investigating this particular group, heterogeneity in the presentation of results and differences in metal concentrations stemming from the point in time when samples are collected. Limiting our comparisons to women at around 38 weeks of pregnancy, lead and cadmium concentrations were also similar to those found in other studies [45,46], while again mercury concentrations were much higher [45].

The positive correlations found for lead and mercury between maternal and cord blood, evidence the diffusion of these metals across the placental barrier. In the case of cadmium, the lower correlations observed reflect the efficiency of the placenta as a barrier against the passage of this metal [14]. Mercury concentrations were higher in newborns than in their mothers. This has been previously reported in the literature [47,48] and attributed to the high affinity of methlymercury to haemoglobin [49], as haemoglobin concentrations are much higher in cord blood than in maternal peripheral blood. Lead and cadmium concentrations were 60% and 54% of the concentration in maternal blood.

Consistent with previous studies [50,51], children from smoking mothers showed higher blood lead and cadmium concentrations, particularly among current smokers. Blood lead levels were also higher in newborns whose mothers were passively exposed to tobacco smoke, supporting the role of passive smoking as an important determinant of blood lead in children [52]. Additionally, umbilical cord lead levels showed seasonal periodicity. Regarding mercury, both maternal and paternal fish consumption were associated with its levels in umbilical cord blood. However, because fish intake was highly correlated among parents, the results observed in fathers probably reflect the underlying association between maternal fish consumption and cord blood mercury. No other studied parental socio-demographic or environmental factors were associated with metal levels in cord blood.

Exposure to heavy metals in women and men of reproductive ages is an issue of current concern, since the evidence is rising that even low levels of exposure to these compounds can affect fetal growth and development [53,54]. Exposure to maternal lead during pregnancy has been proposed as a risk factor for reduced fetal growth [55,56]. However, studies estimating newborn’s exposure through cord blood [53,57-59] have found contradictory results, suggesting that the possible effects of this metal on newborn’s health could depend on the dose [60] or on the timing of exposure [61]. At similar cord blood lead concentrations as those observed in BioMadrid, various reports have also failed to find associations with the studied outcomes [37,60].

Investigations evaluating the possible effects of in-utero exposure to mercury on fetal growth are limited and have yielded inconsistent results [45,62]. Some of these studies have shown an inverse association between cord blood mercury and fetal growth [12,63] or the Apgar scores [60]. Differences between reports have been recently attributed to maternal fish consumption habits, as many species contain beneficial nutrients that can compensate mercury toxic effects [12]. In this regard, a cohort study of pregnant women in England has shown a reverse association between maternal lean and oily fish intake and the risk of intrauterine growth retardation [64], while fatty fish consumption has been related with decreased fetal growth [65]. Unfortunately, we could not evaluate if the results observed in our study varied by type of fish consumed, due to a lack of statistical power. Overall, our findings do not support an association between total mercury in cord blood and fetal health.

Although there is epidemiological evidence that in utero exposure to cadmium may have detrimental effects on newborn’s health [15,16,66-69], the evidence at relatively low levels of exposure as those observed in BioMadrid is scarce. At comparable cadmium levels, studies evaluating the association between cord blood cadmium concentrations and fetal growth have found inconsistent results, with some suggesting possible deleterious effects [66] while others do not [37]. In our study, umbilical cadmium was not associated with fetal growth, but newborns with cadmium concentrations over 0.29 μg/L had lower 1-minute and 5-minute Apgar scores compared to those with lower concentrations. Apgar scoring allows evaluating the condition of infants immediately after birth [70]. Additionally, there is evidence that lower 5-minute Apgar scores at birth may be associated with lower survival and neurological outcomes at one year of age [71] and during early adulthood [72,73]. To our knowledge only two studies have previously evaluated the association between cord blood cadmium concentrations and Apgar scores [60,74]. Mokhtar et al. first reported a negative correlation between umbilical cadmium and the 5-minute Apgar score, in 100 Egyptian newborns with a mean cord blood cadmium level of 0.66 μg/L. Subsequently, a recent study by Al-Saleh et al. based on 1578 babies with a mean cord blood cadmium concentration of 0.78 μg/L showed that newborns with Apgar 5-minutes scores below the 10^th^ percentile (0-8) had higher levels of umbilical cord blood cadmium. Interestingly, our results are consistent with these findings within a substantially lower cadmium concentration range. Although the underlying biological mechanisms explaining this association remain unclear, the potential clinical implications of this finding warrants further evaluation.

Our study has some limitations, including its sample size and the representativeness of the study sample [26]. The decision to study women who intended to give birth in a public hospital implied the exclusion of a proportion of couples with private insurance and, in all likelihood, higher income. However, in Spain there is a National Health System which provides universal and free health care, and the percentage of women in fertile ages who have private insurance is relatively low (around 10% during the study period [75]). Additionally, account must be taken of the fact that the design selected fertile couples in the third trimester of pregnancy, which means that if metals affect fertility or shorten pregnancy length, the concentration in our adult sample may be underestimating the levels in the general population. Another issue of concern is the high percentage of cord blood samples with cadmium values under the detection limit. To solve this problem, we opted for using Tobit regression analysis, a method for modelling continuous variables where a large number of observations are censored at a single value [30,76], which in our case corresponded to the limit of detection. Also, anthropometric measurements were performed only one time on infants, resulting in a potential source of measurement error. However, the complexity of the field work, carried out within the public health system, did not allow for collection of additional measurements. Finally, there is the possibility of residual confounding by factors not completely controlled for by the variables included in the analyses.

Our results reinforce the need to establishing biomonitoring programs in our country, while provide support for tobacco smoke and fish consumption as important sources of heavy metal exposure in newborns. Because of the potential deleterious effects of metal exposure on newborns health, more efforts should be made to recommend mothers to quit smoking and reduce mercury intake by choosing low-mercury fish during pregnancy.

## Competing interests

The authors declare that they have no competing interests.

## Authors’ contributions

Drs. NA, MM, MP, JA, BPG, MC, GLA, AMPM, EG, CP, AI, JCS, AA, AS, JFG, JMGS and JV designed and coordinated the project. Drs. MAF and MJG participated in the design and coordination of the project and also carried out the laboratory analyses. Dr EGE conducted statistical analyses and drafted the manuscript. Dr. PFN provided statistical support. All authors reviewed and approved the final manuscript as submitted.

## Pre-publication history

The pre-publication history for this paper can be accessed here:

http://www.biomedcentral.com/1471-2458/13/841/prepub
